# Coherent magnetic semiconductor nanodot arrays

**DOI:** 10.1186/1556-276X-6-134

**Published:** 2011-02-11

**Authors:** Yong Wang, Faxian Xiu, Ya Wang, Jin Zou, Ward P Beyermann, Yi Zhou, Kang L Wang

**Affiliations:** 1 Materials Engineering and Centre for Microscopy and Microanalysis, The University of Queensland, St Lucia Campus, Brisbane QLD 4072, Australia; 2 Electrical Engineering Department, University of California, Los Angeles, 56-125B Engineering IV Building, Los Angeles, CA, 90095, USA; 3 Department of Physics, University of California-Riverside, 900 University Ave., Riverside, CA, 92521, USA

## Abstract

In searching appropriate candidates of magnetic semiconductors compatible with mainstream Si technology for future spintronic devices, extensive attention has been focused on Mn-doped Ge magnetic semiconductors. Up to now, lack of reliable methods to obtain high-quality MnGe nanostructures with a desired shape and a good controllability has been a barrier to make these materials practically applicable for spintronic devices. Here, we report, for the first time, an innovative growth approach to produce self-assembled and coherent magnetic MnGe nanodot arrays with an excellent reproducibility. Magnetotransport experiments reveal that the nanodot arrays possess giant magneto-resistance associated with geometrical effects. The discovery of the MnGe nanodot arrays paves the way towards next-generation high-density magnetic memories and spintronic devices with low-power dissipation.

## Introduction

Ferromagnet/semiconductor hybrid structures attract great attention as artificial materials for semiconductor spintronics since they have magnetic and spin-related functions and excellent compatibility with semiconductor device structures [1]. By embedding magnetic nanocrystals into conventional semiconductors, a unique hybrid system can be developed, allowing not only utilizing the charge properties but also the spin of carriers, which immediately promises next-generation non-volatile magnetic memories and sensors [2,3]. On the other hand, spin-injections into the semiconductor can be dramatically enhanced via coherent nanostructures, which considerably reduce undesired spin scatterings [4]. Although magnetic hybrid systems, such as MnAs/GaAs, have been extensively studied over several decades, the control (over the spatial location, shape and geometrical configuration) of the magnetic nanostructures (for instance MnAs) still remains a major challenge to further improve the performance of the related magnetic tunnel junctions (MTJs) and spin valves [5]. Here, we report a general and innovative growth approach to produce coherent and defect-free self-assembled magnetic nanodot arrays with an excellent reproducibility in the MnGe system, which reveals a geometry-enhanced giant and positive magnetoresistance (MR). The discovery of the controllable MnGe nanodots with excellent magnetotransport property paves the way towards future magnetoelectronic and spintronic devices with novel device functionalities and low power dissipation. Remarkably, this innovative method can be possibly extended to other similar systems, such as (Ga,Mn)As, (Ga,Mn)N [2], and (Zn,Cr)Te [2,3].

Magnetic semiconductors, making use of both the charge and the spin of electrons, have been studied extensively in the past few years because of their promising applications in spintronic devices [1-11]. Examples of such devices include ferromagnetic heterojunction bipolar transistors, MTJs, magnetically tunable resonant tunneling diodes, magneto-optical modulators, and spin field effect transistors (Spin FETs) [12]. However, the realization of these devices relies significantly on the ability to coherently integrate ferromagnetic materials with semiconductors and effectively control the shape or/and geometrical configuration of the integrated magnetic semiconductors, avoiding undesired spin scatterings, which is extremely crucial for the injection and detection of spin-polarized currents [1,4,12,13]. In pursuit of coherent magnetic/semiconductor systems, previous efforts were predominately devoted to the developments of hybrid ferromagnet/semiconductors, in which epitaxial ferromagnet layers grown on lattice matched semiconductors are desirable to reduce detrimental spin scatterings [1,4,12,13]. As a consequence, a hexagonal (H)-structured MnAs ferromagnet, epitaxially grown on or embedded into the zinc-blende (ZB)-structured GaAs, becomes a promising candidate for spin injection devices. Unfortunately, the difficulty to fabricate a coherent MnAs-based MTJ makes it a challenging task to probe spin injection [13] and also the dislocations or distorted lattices at the H-MnAs/ZB-GaAs interfaces would inevitably degrade the spin-polarization [13,14]. To overcome these problems, a feasible solution is to find a coherent MnAs/GaAs system where the lattices of ZB-MnAs nanocrystals match with the ZB-GaAs matrix [15-17]. Indeed, the coherent ZB-MnAs/ZB-GaAs system can be technically achieved through spinodal decomposition in Mn-doped GaAs. Interestingly, the magnetic and magneto-optical properties of this coherent hybrid ZB-(Ga,Mn)As system are quite different from the H-MnAs/ZB-GaAs system and some exciting phenomena have been observed [16,17]. For instance, the Curie temperature (*T*_c_) has been increased from 313 K (H phase) to 360 K (ZB phase) [16] and a striking memory effect was observed in the system [17]. However, the coherent ZB-MnAs nanocrystals produced by the spinodal decomposition in ZB-(Ga,Mn)As are difficult to control their locations, shapes and geometrical configurations, which has been a major barrier to integrate these hybrid materials in order to make use of their full potentials in spintronic applications and to discover new collective properties from these unique systems [15-17].

Similar to the (Ga,Mn)As system, coherent dopant-rich nanocrystals induced by the spinodal decomposition also exist in most magnetic impurity-doped semiconductor systems, such as MnGe [18-24], (Ga,Mn)N [2], and (Zn,Cr)Te [3]. The common disadvantage of these current available coherent magnetic nanocrystals, as mentioned above, is their random distribution, in terms of size and location, and low controllability. For instance, in the MnGe system, although Jamet et al. [19] recently employed the spinodal decomposition method to fabricate self-organized MnGe nanocolumns with high ferromagnetism, the growth window is narrow and difficult to reproduce. On the other hand, strain fields generated at strained interfaces of two materials with different lattice parameters have been successfully employed to grow quantum dots for several decades [9,10,25-28], underpinning a promising development of high-density three dimensional memories and spatial light modulators for advanced photonic applications [2]. Here, we uniquely combine these two growth strengths (spinodal decomposition and strain field) and, for the first time, demonstrate a general and well-repeatable method to produce coherent and self-organized magnetic nanostructures with superior magnetoresistance in the MnGe system. More strikingly, this innovative method can be easily employed to other diluted magnetic semiconductor systems with spinodal decomposition [2], such as (Ga,Mn)As, (Ga,Mn)N, and (Zn,Cr)Te. Indeed, it is expected to be applicable in any systems where the spinodal decomposition exists.

### Experimental details

#### Growth

**Figure 1:**
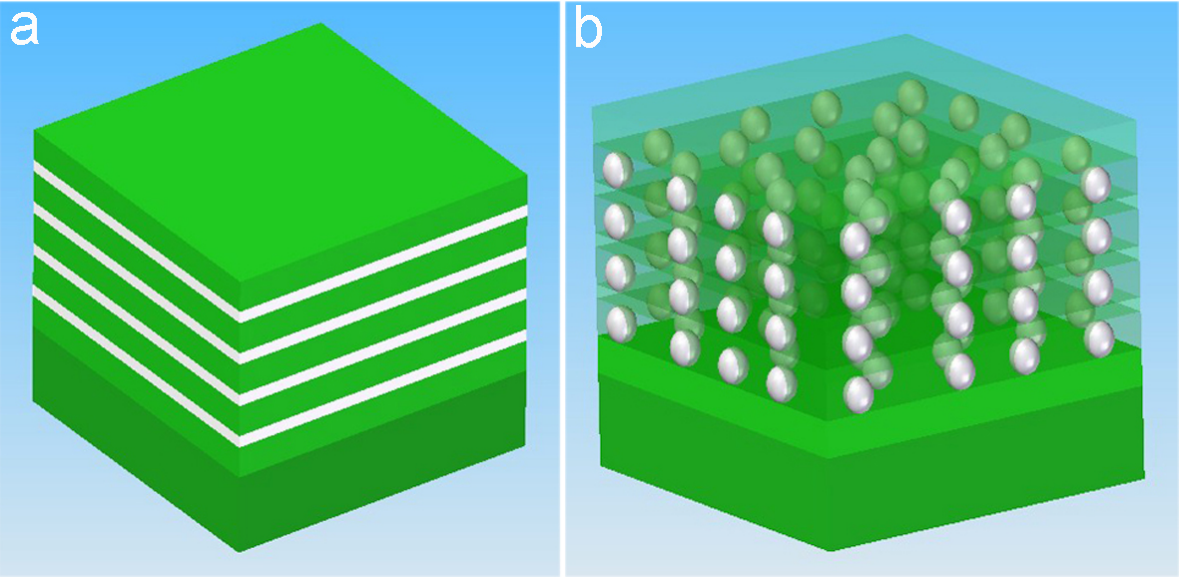
**Schematic drawings of MnGe nanodot arrays**. **(a) **Controlled growth approach of inter-stacked Ge (green) and MnGe (bright) layers with a sequence from the bottom: substrate (Ge or GaAs)/Ge buffer layer/four (MnGe/Ge) layers. **(b) **MnGe nanodot arrays.

A "superlattice" growth approach was carried out by alternating the growth of Mn-doped Ge and undoped Ge thin layers with a Perkin Elmer molecular beam epitaxy. High-purity Ge (99.9999%) and Mn (99.99%) sources were evaporated by conventional high-temperature effusion cells. During the growth, a Ge growth rate of 0.2 Å/s with an adjustable Mn flux as the dopant source was used. The designated structure is schematically shown in Figure 1 and Figure S1 in Additional file 1. First of all, a high-quality single-crystalline Ge buffer layer was deposited at 250°C with a thickness of ca. 60 nm. The surface of the buffer layer was monitored by the reflection high-energy electron diffraction (RHEED) technique and found to be atomic flat evidenced by the streaky RHEED patterns. The growth temperature was then decreased to 70°C for the subsequent "superlattice" growth. Ten periods of Ge and MnGe layers were grown for each case. Different growth parameters (including nominal thicknesses of Ge and MnGe layers, the Mn concentrations and the growth temperatures) were employed in order to obtain MnGe nanodot arrays. It is worthwhile noting that the quality of the buffer layer is crucial for the subsequent low-temperature growth of the MnGe film.

### Structural Characterizations

**Figure 2:**
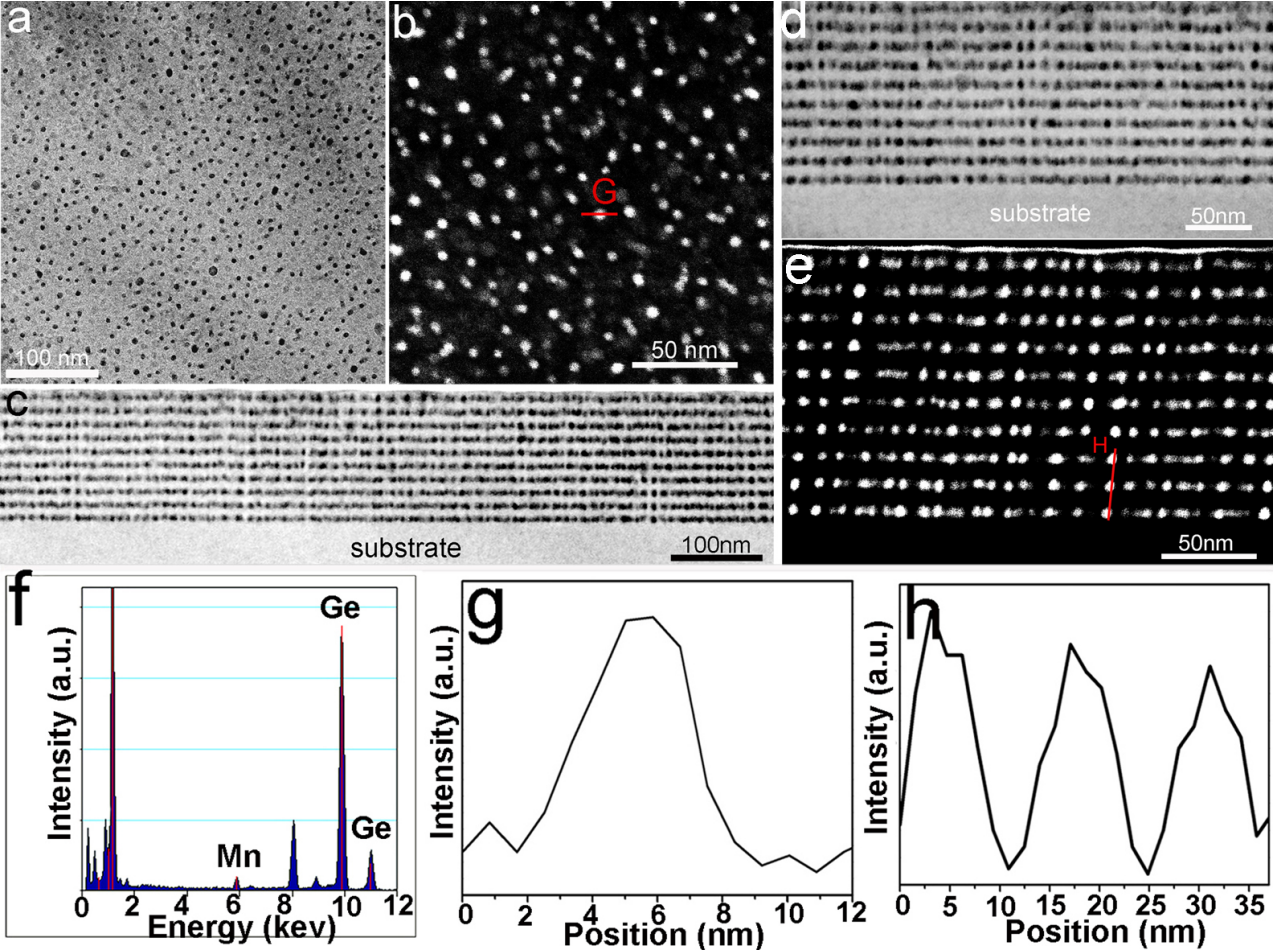
**Transmission electron microscopy (TEM), scanning TEM and energy dispersive X-ray spectroscopy (EDS) results of the multilayer MnGe nanodots**. **(a) **A typical low-magnification plane-view bright-field TEM image showing MnGe nanodots (dark spots). **(b) **A plane-view low-angle dark-field STEM image showing the MnGe nanodots (white spots). **(c) **A low-magnification cross-sectional bright-field TEM image showing the obtained MnGe nanodot array in a large area. **(d) **A higher-magnification cross-sectional TEM image and **(e) **a cross-section STEM image, both showing the MnGe nanodot arrays. (f) A EDS profile showing the Mn and Ge peaks. **(g**, **h) **EDS line-scan profiles of the marked line in (b) and (e) using Mn *K *peak, respectively, confirming nanodots being Mn rich. All TEM images are taken from the same sample.

The high-resolution transmission electron microscopy (TEM) and scanning TEM (STEM) experiments were performed on a FEI Tecnai F20 (S)TEM operating at 200 kV. The digital images were recorded by a Gatan^® ^2k × 2k CCD camera. All the TEM and STEM images were taken in standard conditions. However, it should be noted that the MnGe nanodots appear dark contrast in the bright-field TEM mode (Figure 2a, c, d) which is different from the case in the low-angle dark-field STEM mode (Figure 2b, e) where the MnGe show white contrast due to different imaging systems.

### Property measurements

A physical property measurement system and superconducting quantum interference device was used to measure the magnetotransport and magnetic properties, respectively. Both equipments were manufactured from Quantum Design.

## Results and discussions

### Structural properties

**Figure 3:**
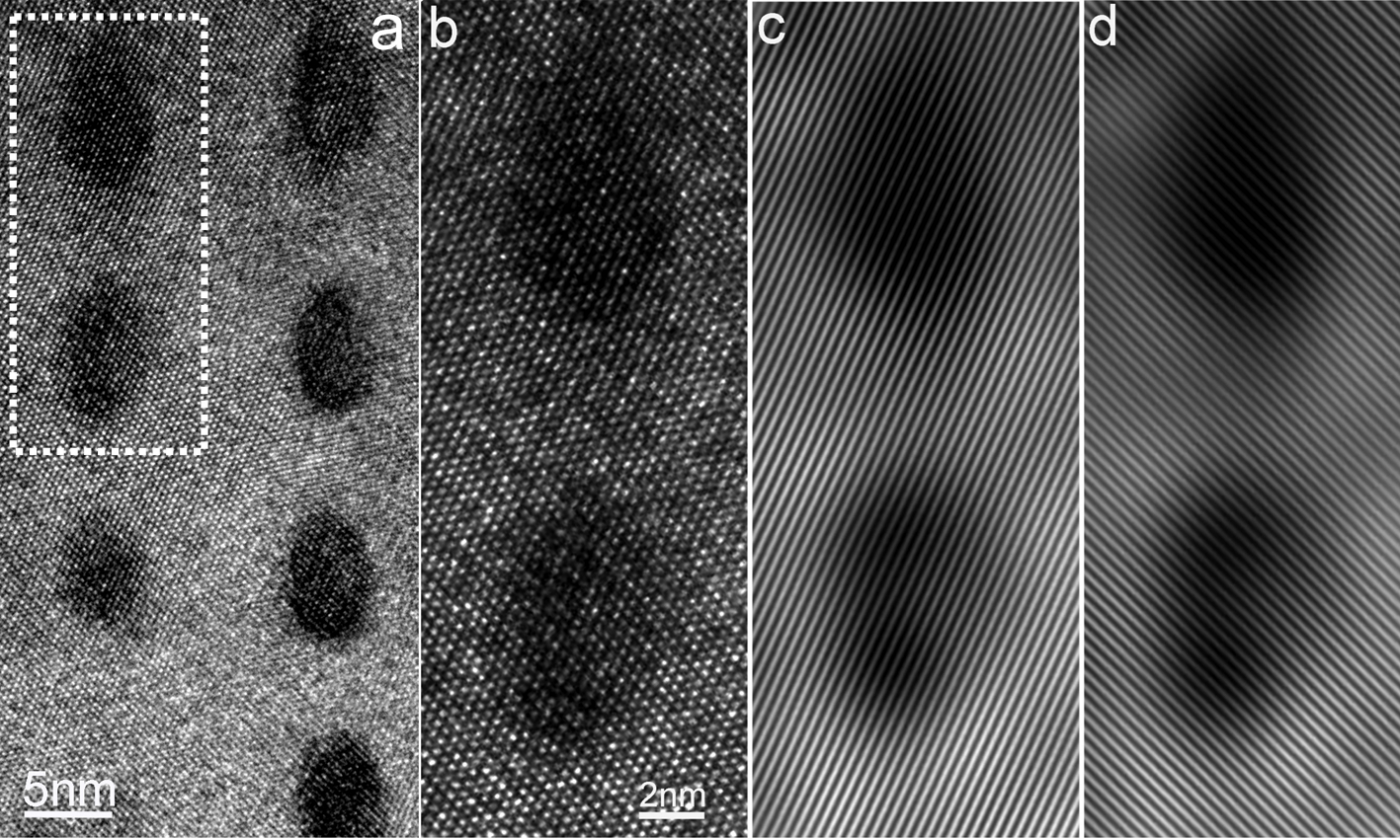
**High resolution transmission electron microscopy results (HRTEM) of the MnGe nanodots**. **(a) **A high-magnification TEM image showing several aligned MnGe nanodots. **(b) **The HRTEM images of the MnGe nanodots (the selected area in (a)) showing a perfect diamond structure as the Ge matrix. **(c**, **d) **Bragg filterings of ± (111) (c) and ± ($1\bar{1}\bar{1}$) (d) reflections, respectively; where no dislocation or distortion was observed. The dark contrast of the nanodots indicates the existence of significant strain.

Practically, we employed a concept of stacked MnGe nanodots by alternatively growing MnGe and Ge layers with designated thicknesses (nominal 3-nm-thick MnGe and 11-nm-thick Ge), as shown in Figure 1a. It is well known that Mn doping in Ge induces compressive strain because of its larger atomic size [29], assuming that no lattice defects are generated during the doping process, i.e., lattice coherence. Mn-rich MnGe nanodots induced by the spinodal decomposition should be strained if the lattice coherence between the nanodots and the matrix remains. Once the strained nanodots are developed, a thin Ge spacer layer, subsequently deposited with an optimized thickness, will retain the perfect lattice coherence with the underneath nanodots. This enables the existing nanodots to exert strain on the Ge spacer layer and produce "strained spots", which, in turn, become preferred nucleation sites for successive nanodots. Eventually, multilayered and vertically aligned MnGe nanodot arrays can be produced, similar to the scenarios of stacked InAs/GaAs [27,28] and Ge/Si [30] quantum dots. Indeed, by employing this innovative approach, we achieved the growth of coherent self-assembled MnGe nanodot arrays with an estimated density of 10^11 ^cm^-2 ^to approximately 10^12 ^cm^-2 ^within each MnGe layer, as schematically demonstrated in Figure 1b. In this study, ten periods of MnGe nanodots were epitaxially grown on Ge (100) and GaAs (100) substrates by a Perkin-Elmer solid source molecular beam epitaxy (MBE) system. A detailed description of growth method and parameters are presented in the Methods part (also refer to Figure S1 in Additional file 1). TEM and energy dispersive spectroscopy (EDS) in the STEM mode were performed to understand the nanostructures and compositional variations of the resulting thin films. Figure 2a and 2c are typical plane-view and cross-sectional TEM images and show the general morphology of the MnGe nanostructures, viewed along the <100> and <011> directions, respectively. A high-density of dark nanodots can be clearly seen in both cases. Based on the magnified cross-sectional image shown in Figure 2d, the nanodot arrays are clearly observed with ten stacks along the growth direction although not perfectly vertical (see Figure S2 in Additional file 1 for more images). In order to determine the composition of the dark dots, EDS analyses in the STEM mode were carried out and typical plane-view and cross-sectional STEM images are shown in Figure 2b and 2e, respectively. Figure 2f is the EDS result taken from a typical dot and shows clearly the Mn and Ge peaks. Figure 2g and 2h present EDS line scans using the Mn *K *peak for the dots marked by G and H in Figure 2b and 2e, respectively, indicating high concentrations of Mn inside the dots. Taking all these comprehensive TEM results into account, it is concluded that the nanodots are Mn-rich when compared with the surrounding matrix. Figure 3a shows a high magnification TEM image taken from a thin area, where several aligned MnGe nanodots can be evidently observed. The distance between two vertically adjacent nanodots (along the growth direction) is measured to be 14 ± 1 nm, well matched with the designed period of a 11-nm-thick Ge spacer layer and a 3-nm-thick MnGe layer. It should be noted that these nanodots are uniform in size with an elliptical shape (a dimension of 5.5 ± 0.5 nm and 8 ± 0.3 nm in the horizontal and vertical directions, respectively), as demonstrated in Figure 3a. Since the nominal thickness of the MnGe layer (3 nm) is far less than the dot vertical dimension (8 nm), it suggests that, during the growth of the MnGe thin film, Mn not only diffuses laterally (to form dots), but also migrates vertically into the adjacent Ge spacer layers, primarily in the proximity of the dot regions, resulting in ellipse-shaped nanodots.

To determine structural characteristics of the MnGe nanodots at the atomic level, high-resolution TEM (HRTEM) was used and an example is shown in Figure 3b, where the HRTEM image was taken from the dashed rectangle area in Figure 3a. Interestingly, a careful examination of the HRTEM image shows that the MnGe nanodots have an identical single-crystalline structure to the Ge matrix (the diamond structure) with no observed lattice defects, consistent with other reports (with irregular shape of MnGe clusters) [18,19]. As mentioned above, this type of MnGe nanodots is lattice coherent. This is substantially different from other Mn-rich precipitates such as hexagonal Mn_5_Ge_3 _[31] and Mn_11_Ge_8 _[32] which have a different phase, other than a diamond structure as Ge matrix. This is also verified by our selected area diffraction patterns (refer to Figure S2 in Additional file 1), where no extra diffraction spots or diffused ring(s) can be observed. To further determine the possible lattice distortion of the MnGe nanodots with respect to the Ge matrix, the inversed Fourier transform (Bragg filtering) technique [19] was used where two sets of nano atomic planes are shown in Figure 3c and 3d. As can be observed, the interfaces between the MnGe nanodots (the dark areas) and the Ge matrix are perfectly coherent without noticeable lattice distortion or bending of the atomic planes. In fact, using the (111) atomic spacings away the Ge matrix as a reference, the MnGe spacing of (111) atomic planes are determined to be identical to that of the Ge matrix. A quantitative EDS analysis suggests that the dots have a Mn concentration as high as 11% (Figure 2f), which can be further adjusted by altering the Mn flux during the growth. The high Mn doping is comparable to the reported Mn concentration of 15% in Ref. [18]. Since the atomic radius of Mn (140 pm) is larger than that of Ge (125 pm) [29], it is expected that these Mn-rich dots experience a compressive stress caused by the surrounding Mn-poor Ge matrix. In fact, such a stored stress can be visualized from the strong contrast of the nanodots shown in Figure 3. Therefore, the successful vertical alignment of stacked nanodots can be attributed to the strain fields induced by the underlying Ge spacer layers, which is consistent with the growth mechanism of stacked quantum dot systems.

### Magnetic properties

Since the nanodot array samples are ferromagnetic below 300 K (Figure S3 in Additional file 1), it is of great interest to study their magnetotransport properties. To do this, the samples were then fabricated into standard Hall bars with a typical channel width of 500 μm. For all measurements, the external magnetic field (*H*) was applied perpendicular to the sample surface. In order to completely avoid the substrate (Ge) conducting effect (Figure S4 in Additional file 1) [33], we have also successfully grown the same nanostructures on GaAs substrates under the same growth conditions as GaAs has the almost identical lattice parameter as Ge.

**Figure 4:**
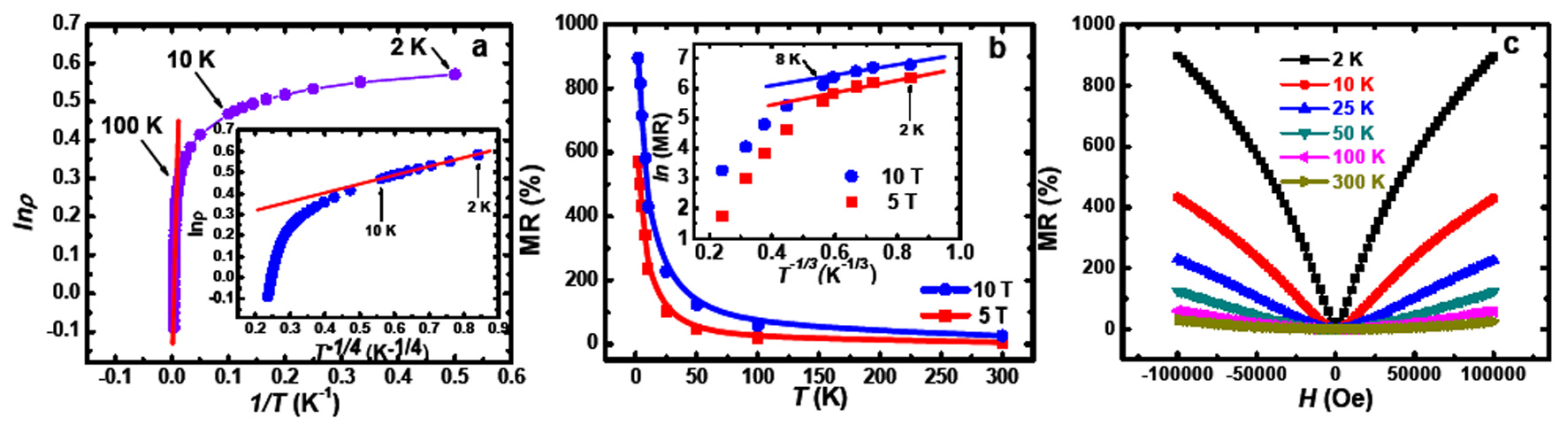
**Magnetotransport measurements for the MnGe nanodot arrays**. **(a) **the temperature-dependent resistivity (ln*ρ *versus *T*^-1^) and the inset displays the plot of ln*ρ *versus *T*^-1/4^. **(b) **Temperature-dependent MR under fixed magnetic fields of 5 and 10 Tesla and the inset showing the plot of ln(MR) vs *T*^-1/3^. **(c) **Positive MRs at different temperatures and different magnetic fields.

The resistivity measurements were carried out to probe the carrier transport under different temperatures. It was found that the temperature-dependent resistivities rapidly increase with decreasing temperature due to the carrier freeze-out effect at low temperatures, which is typically observed in doped semiconductors [34]. Considering the embedded MnGe nanodots, the rise in resistivities at low temperatures also suggests a strong localization of carriers, which takes place at the Mn sites and/or at the MnGe/Ge interfaces, similar to the scenario of MnSb clusters in InMnSb crystals [8]. The temperature-dependent resistivity can be generally described by [35]

(1) $$\rho (T)=\rho_{0}\exp[{\left(\frac{T_{0}}{T}\right)}^{1/\alpha }],$$

where *ρ*(*T*) is the temperature-dependent resistivity; *ρ*_0 _and *T*_0 _denote material parameters, *α *is a dimensionality parameter: *α *= 2 for one-dimensional (1D), *α *= 3 for 2D, and *α *= 4 for 3D systems. In order to reveal the carrier transport mechanisms at different temperature regions, fittings were performed in the plots of lnρ as a function of *T*^*-α *^(Figure 4a). The best fittings were found when *α *equals to 1 and 4 in the high-temperature and low-temperature regions, respectively, corresponding to the carrier transport via the band conduction [36] (thermal activation of acceptors) and the 3D Mott's variable range hopping processes [35]. According to the fitting results to Equation 1, the obtained nanodot arrays show a dominated hopping process below 10 K. At such a low temperature, the majority of free holes are recaptured by the acceptors. As a result, the free-hole band conduction becomes less important and hole hopping directly between acceptors in the impurity band contributes mostly to the conductivity [36]. Above 100 K, the conduction is dominated by the thermal activation of the holes (the band conduction). A thermal activation energy (*E*_*a*_) of 15 meV can be obtained from Equation 1 with *α *= 1 and *E*_*a *_= *T*_0_*K*_*B*_, where *K*_*B *_is the Boltzmann constant. This activation energy does not correspond to any known acceptor energy levels due to Mn doping in Ge, consistent with results shown in reference [20].

To explore practical applications for our extraordinary nanodot arrays, the MR measurements were performed from 2 to 300 K with an external magnetic field up to 10 Tesla. Figure 4b shows the plots of temperature-dependent MR at given magnetic fields (5 and 10 Tesla) for the nanodot arrays. Under a strong magnetic field, the MR in the region of variable range-hopping conduction can be described by [37,38]

(2) $$\text{MR}(H)=\exp[\frac{C}{{\left(\lambda^{2}T\right)}^{1/3}}]-1,$$

where the magnetic length *λ *equals to (*cħ*/*eH*)^1/2 ^and C is a field and temperature independent constant. Note that the Equation 2 is only valid in a strong-field limit [37-39]. The inset in Figure 4b shows the best fitting results, in which a linear behavior of MR versus *T*^-1/3 ^is obtained, further confirming the hopping conduction mechanisms (*T *≤ 8 K). Note that the absolute values of MRs were used for the fitting purpose. These fitting results are reasonably close to the obtained hopping regions determined from the zero-magnetic-field resistivity measurements (*T *≤ 10 K, Figure 4a).

It is striking to observe that the coherent MnGe nanodot arrays present a large and positive MR up to 900% at 2 K (Figure 4c). Traditionally, the positive MR is attributed to the Lorentz force in the semiconductor matrix, which deflects the carriers during the transport process [39]. The resulting MR is positive and proportional to (*μH*)^2 ^under low magnetic fields [19] (*H *≤ 1 Tesla in our case) where *μ *is the semiconductor mobility (units *m*^*2*^*V*^*-1*^*S*^*-1 *^or *T*^*-1*^) and *H *is the magnetic field. However, with a simple calculation, the estimated orbital MR is too small to explain the large MR observed from the nanodot arrays. Instead, we anticipate that besides the effect of orbital MR, the high-density magnetic nanodots could significantly contribute to the large MR ratios due to an enhanced geometric MR effect, from which the current path may be significantly deflected when external magnetic fields were applied to the magnetic nanostructures [19,40,41]. To elucidate the underlying physics of the geometrical effect, we consider a thin Hall bar geometry with a measurement current applied in the *x*-direction, a Hall voltage in the *y *direction, *z *direction normal to the sample surface, and an external magnetic field *H *parallel to *z*. For semiconductors, the current density and the total electric field can be described by $j=\bar{\sigma }E$, where the magneto-conductivity tensor is given by [40,41]

(3) $$\bar{\sigma }(H)=\left(\begin{array}{ccc} \frac{\sigma }{1+\beta^{2}} & \frac{\sigma \beta^{2}}{1+\beta^{2}} & 0\\ \frac{-\sigma \beta^{2}}{1+\beta^{2}} & \frac{\sigma }{1+\beta^{2}} & 0\\ 0 & 0 & \sigma \end{array}\right).$$

Here, *β *= *μH*. At zero magnetic field, *β *vanishes. The conductivity tensor is diagonal when lacking of the magnetic field; and the current density can be simply described by *j *= *σE*. Since the electric field is normal to the surface of a metallic inclusion and *j *|| σ*E *, the current flowing through the material is concentrated into the metallic region which behaves like a "short circuit" (Figure S5 in Additional file 1) [42]. As a result, the inclusion of metallic clusters can lead to a higher conduction than that of a homogeneous semiconductor [19,40,41]. However, at high magnetic fields (*β*>>1), the off-diagonal terms of $\bar{\sigma }(H)$ dominate. Equivalently, the Hall angle between *j *and *E *approaches 90°(*j*⊥*E*); and the current becomes tangent to the nanodots. This further indicates that the current is deflected to flow around the nanodots, resembling an "open circuit" state (Figure S5b in Additional file 1) [42]. The transition from the "short circuit" at the zero field to the "open circuit" at high fields produces an increase of resistance, i.e., a positive geometrically-enhanced MR [41]. The above explanation has been successfully applied to several material systems, including Au/InSb [41] and MnAs/MnGaAs [42]. Similarly, the geometrically-enhanced MR (ca. 200% at 10 Tesla, 300 K) was identified in MnGe_2 _nanostructures with a high Mn concentration of approximately 33% [19].

## Conclusion

In conclusion, we have successfully developed a novel approach to fabricate extraordinarily coherent and self-organized MnGe nanodot arrays embedded in the Ge and GaAs matrixes by low-temperature MBE. A high yield of such aligned nanodot arrays was confirmed on different substrates, showing an ideal controllability and reproducibility. More importantly, giant positive magneto-resistances were obtained due to the geometrically-enhanced effect. We anticipate that our studies will advance the development of MnGe magnetic semiconductors and/or other similar systems. The obtained coherent and self-assembled nanostructures could be potentially used as the building blocks in the high-density magnetic memories, sensors and spintronic devices, enabling a new generation of low-dissipation magnetoelectronic devices.

## Competing interests

The authors declare that they have no competing interests.

## Authors' contributions

YW, FX conceived and designed the experiments. YW, FX carried out the experiments with contribution from YW, YZ and WB. KW and JZ supervised the work. FX, YW, JZ, and KW wrote the manuscript. All authors read and approved the final manuscript.

## Open Access

This article is distributed under the terms of the Creative Commons Attribution Noncommercial License which permits any noncommercial use, distribution, and reproduction in any medium, provided the original author(s) and source are credited.

## Supplementary Material

Additional file 1 **Supporting Figures S1-S5**. Figure S1. A schematic drawing of the growth architecture; Figure S2: A demonstration of excellent reproducibility; Figure S3. Magnetic properties of the MnGe nanodot arrays; Figure S4. Magnetoresistance for the MnGe nanodot arrays and Ge substrates; Figure S5. Models of magneto-transport for the MnGe nanodot arrays. Click here for file
