# Supplementary material for: Coherent magnetic semiconductor nanodot arrays
Source: Nanoscale Res Lett. 2011 Feb 11;6(1):134. doi: 10.1186/1556-276X-6-134 (PMC3211181; doi:10.1186/1556-276X-6-134)
Supplement: Additional file 1 — Supporting Figures S1-S5. Figure S1. A schematic drawing of the growth architecture; Figure S2: A demonstration of excellent reproducibility; Figure S3. Magnetic properties of the MnGe nanodot arrays; Figure S4. Magnetoresistance for the MnGe nanodot arrays and Ge substrates; Figure S5. Models of magneto-transport for the MnGe nanodot arrays. [file 1556-276X-6-134-S1.PDF]

Figure S1. A schematic drawing of the growth architecture

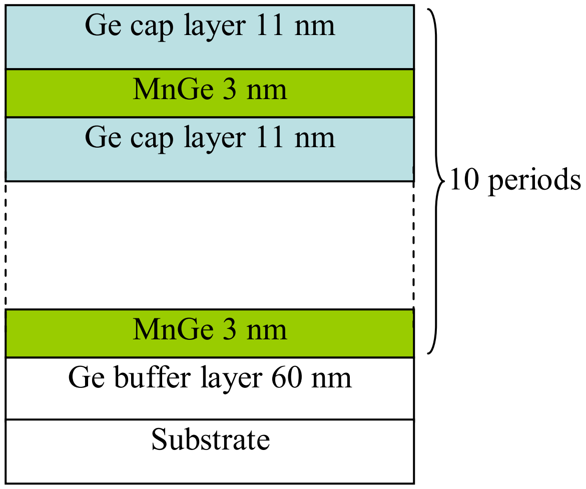

Figure S1. A schematic drawing of the growth strategy designated as "superlattice growth method". The MnGe and Ge layers are alternatively grown on a Ge buffer layer. The drawing gives a typical set of growth parameters to produce MnGe nanodot arrays. Parameters, such as growth temperature, the thickness of MnGe and Ge, and the Mn concentration, can be altered in order to achieve various nanostructures, for example, nanocolumns and nanowells.

Figure S2: A demonstration of excellent reproducibility

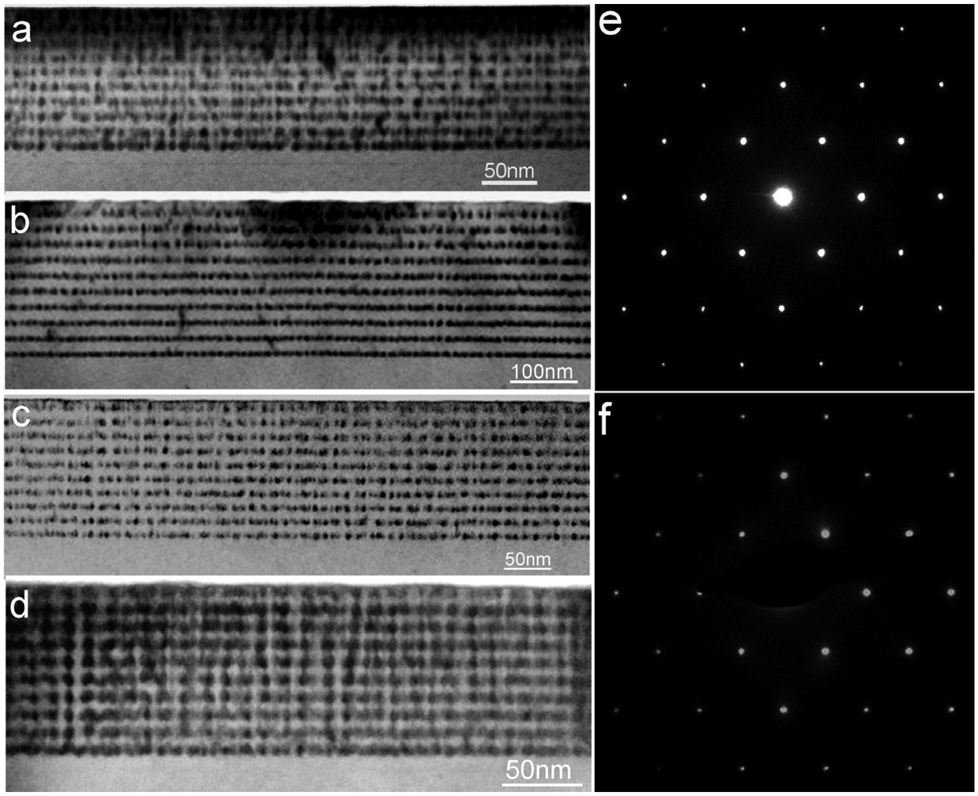

Figure S2. A demonstration of excellent reproducibility of MnGe nanodot arrays by using the parameters shown in Figure S1. Selected area electron diffraction patterns taken from these samples show a diamond structure.

Figure S3. Magnetic properties of the MnGe nanodot arrays

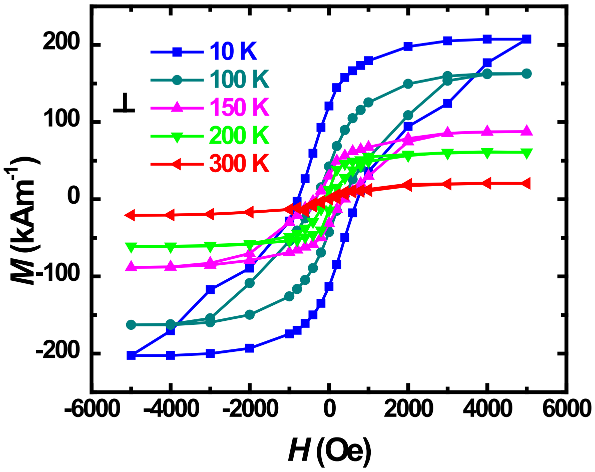

Figure S3. Temperature dependent hysteresis loops with the sample surface perpendicular to the external magnetic field.

Figure S4. Magnetoresistance for the MnGe nanodot arrays and Ge substrates

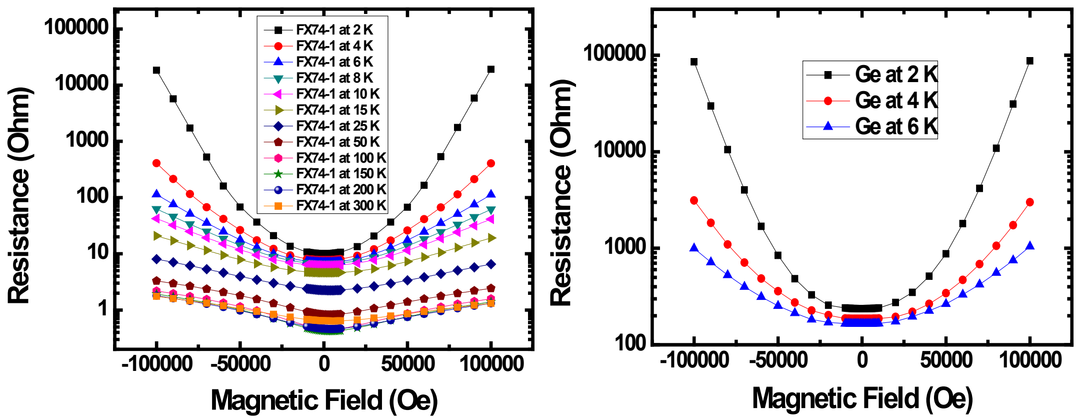

Figure S4. (a), Magnetoresistance (MR) for MnGe nanodot arrays grown on conducting Ge substrate; (b), MR for the conducting Ge substrate. These results indicate that the conducting Ge substrate has a significant effect on the MR measurements. Therefore, we use semi-insulating GaAs substrate to exclude this effect.

Figure S5. Models of magneto-transport for the MnGe nanodot arrays

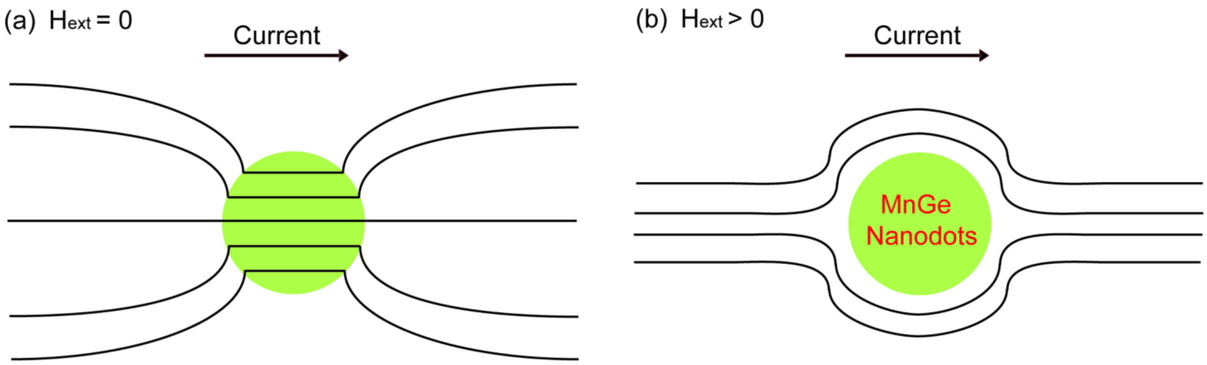

Figure S5. Schematics of carrier transport with and without the external magnetic fields. (a) and (b) resemble short and open circuits, respectively. The model is cited from: Heimbrodt, W. et al. Magnetic interactions in granular paramagnetic-ferromagnetic GaAs:Mn/MnAs hybrids. *J. of Superconductor: Incorporating Novel Magnetism* **18**, 315 (2005); and Solin et al. Enhanced room-temperature geometric magnetoresistance in inhomogeneous narrow-gap semiconductor, *Science* **289**, 1530 (2000).
